# The impact of climate change on the distribution of rare and endangered tree *Firmiana kwangsiensis* using the Maxent modeling

**DOI:** 10.1002/ece3.9165

**Published:** 2022-07-29

**Authors:** Xiaoxuan Gao, Jing Liu, Zhihuan Huang

**Affiliations:** ^1^ College of Architecture and Design University of South China Hengyang China; ^2^ School of Life Sciences Central China Normal University Wuhan China

**Keywords:** climate change, *Firmiana kwangsiensis*, habitat, plant conservation, predictive model

## Abstract

The upsurge in anthropogenic climate change has accelerated the habitat loss and fragmentation of wild animals and plants. The rare and endangered plants are important biodiversity elements. However, the lack of comprehensive and reliable information on the spatial distribution of these organisms has hampered holistic and efficient conservation management measures. We explored the consequences of climate change on the geographical distribution of *Firmiana kwangsiensis* (Malvaceae), an endangered species, to provide a reference for conservation, introduction, and cultivation of this species in new ecological zones. Modeling of the potential distribution of *F. kwangsiensis* under the current and two future climate scenarios in maximum entropy was performed based on 30 occurrence records and 27 environmental variables of the plant. We found that precipitation‐associated and temperature‐associated variables limited the potentially suitable habitats for *F. kwangsiensis*. Our model predicted 259,504 km^2^ of *F. kwangsiensis* habitat based on 25 percentile thresholds. However, the high suitable habitat for *F. kwangsiensis* is only about 41,027 km^2^. *F. kwangsiensis* is most distributed in Guangxi's protected areas. However, the existing reserves are only 2.7% of the total suitable habitat and 4.2% of the high suitable habitat for the plant, lower than the average protection area in Guangxi (7.2%). This means the current protected areas network is insufficient, underlining the need for alternative conservation mechanisms to protect the plant habitat. Our findings will help identify additional *F. kwangsiensis* localities and potential habitats and inform the development and implementation of conservation, management, and cultivation practices of such rare tree species.

## Graphical abstract

Based on 30 effective occurrence records and 27 environmental variables, we modeling the potential distribution of F. kwangsiensis under current and two future climate scenarios in maximum entropy, to identify the localities where F. kwangsiensis may exist but not yet been detected, and to recognize of localities where it is likely to spread to. Our study provides important information for the conservation management and cultivation of such rare tree species, may generate extensive interests to plant scientists, whom concerned about wildlife conservation, and horticulture lovers.

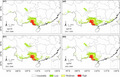

## 1 INTRODUCTION

A comprehensive understanding of the relationship between the geographical distribution of a species and climatic factors is a critical ecological aspect for governments and environmentalists (Lawler et al., 2009; Stocker et al., 2013), because climate change strongly impacts the geographical distribution of species, affecting ecosystems and human well‐being (Du et al., 2021; Klein et al., 2008; Lawler et al., 2009; Smeraldo et al., 2021; Stocker et al., 2013; Tilman & Lehman, 2001; Zhang et al., 2018). Rare and endangered plants, one of the most important biodiversity elements, play an important role in the ecosystem, healthcare, and scientific research roles and possess economic and cultural values (Klein et al., 2008; Okigbo et al., 2008). However, the threat of climate change and anthropogenic disturbances have caused a decline in populations and extinctions in worst cases (Clavel et al., 2011; Oliver et al., 2015; Oliver & Morecroft, 2014). Therefore, evaluating the effect of climate change on the spatial distribution of rare and endangered species reveals optimal conservation and effective management practices in protecting the most endangered species (Qin, Liu, et al., 2017; Qin, Zhao, et al., 2017).

The spatial distribution of rare and endangered plants is usually limited by their inherent characteristics and influenced by human activities. For example, increasing evidence shows that the low genetic diversity may be the main reason for the poor adaptability and narrow distribution of some rare and endangered plants (Abeli et al., 2019; Kyriazis et al., 2020). Habitat fragmentation results from excessive exploitation and utilization of natural resources, which may cause the extinction of some flora and fauna (Fischer & Lindenmayer, 2007; Qiu & Fu, 2001). The spatial distribution of rare and endangered plants could also result from dispersal limitation and demographic stochasticity (Aiba et al., 2012; Clark et al., 2018; Hubbell, 2001; Legendre et al., 2009). Early research has revealed that most endemic species are spatially aggregated due to their short‐ranged dispersal (Clark et al., 2018; Condit et al., 2000; Krebs, 2001). Nevertheless, few studies have distinguished the relative contributions of environmental variables in the distribution of rare and endangered plants.

Species distribution models (SDMs) can predict the geographic distribution of individual species using local data and ecological variables (Franklin & Miller, 2009). Maxent (Maximum Entropy) is one of the powerful tools for modeling endemic species with narrow habitat ranges and few available presence‐only occurrence data (Ancillotto et al., 2020; Elith et al., 2006; Kong et al., 2021; Phillips et al., 2006; Segal et al., 2021; Thapa et al., 2018). Maxent has been used in numerous studies to predict the potential distribution areas of rare and endangered species. For example, Qin, Liu, et al. (2017); Qin, Zhao, et al. (2017) used this model to predict the potential distributions of *Thuja sutchuenensis*, a rare tree species, under paleoclimate, current climate, and future climate. Saputra and Lee (2021) constructed Maxent models for current and potential future habitats for *Styrax sumatrana*. Kong et al. (2021) performed similar modeling for *Osmanthus fragrans*. Maxent modeling can also inform conservation efforts or predict future biodiversity patterns under the then climate (Algar et al., 2009; Distler et al., 2015).

*Firmiana kwangsiensis* (Malvaceae) is a precious tree endemic to Guangxi, South China, but it can grow in Guangdong and Yunnan provinces. This tree species can grow in moist, well‐drained, or limestone soils and tolerate several soil types (Luo et al., 2011). The beautiful shape, large three‐ to five‐lobed leaves, and orange‐red flowers make it an excellent landscaping tree species. In addition, due to its superior sonic properties, the wood is utilized for the soundboards of several Chinese instruments, including *Guqin* and *guzheng*. Ancient evidence indicates the Chinese people have used gelatin from this tree and closely related species for hair conditioning or binding since the Yuan dynasty (Yen, 1956). However, *F. kwangsiensis* is almost extinct due to its weak natural regeneration ability, the effects of tourism, and excessive exploitation, among other reasons. The International Union for Conservation of Nature (IUCN) has listed the plant as a “critically endangered” species (Qin, Liu, et al., 2017; Qin, Zhao, et al., 2017), and it is one of the most protected wild plants by the Chinese government (Lu et al., 2021). The geographical distribution and ecological requirements of *F. kwangsiensis* are largely unknown. Understanding the environmental factors influencing the distribution of *F. kwangsiensis* can reveal the optimal conservation measures for this species.

This study aimed (a) to predict the potential ecological distribution of *F. kwangsiensis* in contemporary, (b) to determine the significant environmental factors affecting the distribution of *F. kwangsiensis*, (c) to predict the potential future distribution of *F. kwangsiensis* under the then climatic conditions, and (d) to recommend priority areas for future effective conservation of *F. kwangsiensis*. The findings of this study revealed the geographical distribution of *F. kwangsiensis*, its potential habitats, the priority selection area for conservation, and methods of introducing and cultivating the tree in new habitats.

## 2 MATERIAL AND METHODS

### 2.1 Species occurrence data collection

**FIGURE 1:**
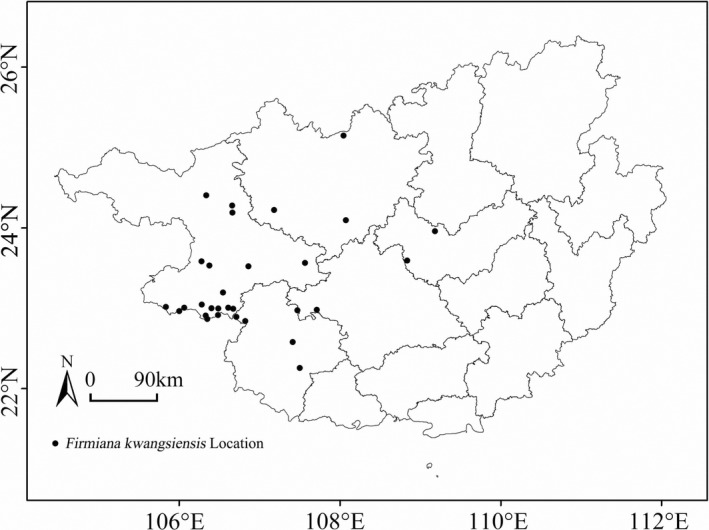
The 30 *Firmiana kwangsiensis* distribution areas.

The geographical distribution of *F. kwangsiensis* in Guangxi, South China, was assessed from May 2016 to July 2018 under the “Investigation and Germplasm Preservation of endangered Wild Plant species in Southwest China” project sponsored by the Ministry of Science and Technology of China. The geo‐reference of all the 67 natural populations and isolated *F. kwangsiensis* individuals in Guangxi was recorded. The occurrence data of the tree species were organized and cleaned to remove repeating coordinates. Data for 30 *F. kwangsiensis* in different geographical locations were incorporated in the final analysis and plotting of the distribution map (Figure 1).

### 2.2 Environmental variables

Temperature, rainfall, geographical barriers, and other ecological factors, such as geological formations, influence species distribution (Kaeslin et al., 2012). To determine the environmental factors that influence the distribution of *F. kwangsiensis*, 19 bioclimatic variables, three biophysical variables (elevation, slope, and aspect), two ground cover factors (ground cover type and vegetation coverage rate), and three human factors (human footprint, human impact, and population density) were included in our model. The current bioclimatic factors and elevation data during 1970–2000 and the future bioclimatic data of two shared socioeconomic pathways (SSP) for predicting *F. kwangsiensis* distribution in 2021–2040 and 2041–2060 were downloaded from the World Climate Database WorldClim2.1 (https://www.worldclim.org/). A 2.5 min (about 4.5 km × 4.5 km) coordinate system (WGS1984) was used. Slope and aspect data were extracted from the global DEM database (http://www.gscloud.cn/). The ground cover type and vegetation coverage data with a resolution of 1 km were obtained from the International Steering Committee for Global Mapping (ISCGM; https://www.iscgm.org/). Data for human factors with a resolution of 1 km were obtained from NASA Socioeconomic Data and Applications Center (SEDAC; http://sedac.ciesin.columbia.edu). The population density data was downloaded from Gridded Population of the world database v4 (GPWv4; https://sedac.ciesin.columbia.edu/data/collection/gpw‐v4). The above data were converted into “.asc” files required by Maxent modeling using the ArcGIS software.

To reduce multicollinearity among the 27 environmental variables, highly correlated variables (*r* ≥ .80 Pearson correlation coefficient) were eliminated from further models (Graham, 2003). Finally, 12 statistical and biologically meaningful variables were used to model the geographical distribution for *F. kwangsiensis* (Table 1). These variables included precipitation in the warmest quarter (BIO18), precipitation in the driest quarter (BIO17), Population density in 2000 (2000), annual precipitation (BIO12), temperature seasonality (BIO4), mean temperature of the wettest quarter (BIO8), minimum temperature of the coldest month (Bio6), percent tree cover (PTC), land cover (LC), slope, human footprint (HF), and aspect.

**TABLE 1:** The significance of environmental variable used for evaluating the potential distribution of *Firmiana kwangsiensis* in China. The relative importance of environmental variables was evaluated using the jackknife test.

Code	Environment variables	Percent contribution (%)	Permutation importance (%)
BIO18	Precipitation of Warmest Quarter	57.5	29.0
BIO17	Precipitation of Driest Quarter	16.8	2.2
2000	Population density in 2000	6.0	0.3
BIO12	Annual Precipitation	5.7	8.2
BIO4	Temperature Seasonality (standard deviation ×100)	4.5	0.5
BIO8	Mean Temperature of Wettest Quarter	3.1	0.2
BIO6	Min Temperature of Coldest Month	2.4	59.1
PTC	Percent Tree Cover	2.3	0.1

### 2.3 Species distribution modeling

The potential habitat of *F. kwangsiensis* were mapped using the maximum entropy model (Maxent version 3.4.1). In the modeling, 75% of the data was used for model training, whereas 25% of the data was used for model testing (Phillips, 2006) while keeping other values as default. Jackknife analyses were performed to determine variables that significantly influence the model reliability. The model's accuracy was assessed based on the area under the receiving operator curve (AUC). The value of AUC ranges from 0 to 1. An AUC value of 0.50 indicates better performance than random, whereas a value of 1.0 indicates perfect discrimination (Swets, 1988). The model performance was therefore directly proportional to the AUC value. For display and further analysis, the Maxent model results predicting the presence of *F. kwangsiensis* (0–1 range) distribution were inputted into the ArcGIS software, V. 10.2. Referring to the classification proposed by Thapa et al. (2018), four habitat classes were generated: unsuitable habitat (0–0.20); lowly suitable habitat (0.20–0.50); moderately suitable habitat (0.50–0.70); highly suitable habitat (>0.70). The area for optimal distribution, classified as low, medium, and high suitable habitats, was calculated for each model.

The contributions of anthropogenic activities and climate change to species distribution were assessed using the multi‐model ensemble simulations from phase 6 of the Coupled Model Intercomparison Project (CMIP6; Eyring et al., 2016). Here, two future scenarios were examined (Table 2) and incorporated into the Shared Socioeconomic Pathways (SSPs; O'Neill et al., 2016). It describes the alternative evolution of the future population in the absence or presence of climate change and its relationship to the Representative Concentration Pathways (RCPs; Van Vuuren et al., 2011). For example, SSP126 combines SSP1‐a further pathway with low challenges for adaptation and mitigation, and RCP2.6‐a further pathway with efficient energy use and low carbon emissions, whereas in SSP585, both challenges are high. Model projections under SSP126 and SSP585 scenarios in CMIP6 were used for mapping potential *F. kwangsiensis* habitat in the 2021–2040 and 2041–2060 periods.

**TABLE 2:** The suitable contemporary habitat (km^2^) for *Firmiana kwangsiensis* and the suitable area under two climate scenarios in the future.

Major classification	Contemporary (1970–2000)	SSP126 (2020–2040)	SSP126 (2041–2060)	SSP585 (2020–2040)	SSP585 (2041–2060)
Low	161,170	136,546	187,394	167,670	122,553
Moderate	57,308	49,997	59,211	63,808	50,726
High	41,027	37,301	43,193	37,199	35,660
Total	259,504	223,844	289,798	268,677	208,940

The SDM toolbox (including present and future SDMs) was used for calculating changes in the distributions of the above SDMs (Kong et al., 2021; Zhang et al., 2018) and the primary shifts in the *F. kwangsiensis* distribution. The species distributions were then reduced to one individual centroid point. The magnitudes and directions of the time‐dependent estimated variations were created. The movements of the centroids of various SDMs were tracked to examine shifts in *F. kwangsiensis* distribution.

### 2.4 Conservation assessment

The existing nature reserves (including national, provincial, and county nature reserve) data for China were downloaded from the National Specimen Information Infrastructure (http://www.nsii.org.cn). To assess the conservation status of *F. kwangsiensis*, we overlaid existing nature reserves with habitat suitability class and calculated the percentages of the area included in the nature reserves (Bosso et al., 2016; Cahyaningsih et al., 2021). Priority conservation areas were identified based on the visual observation of the prediction map as highly suitable habitat that does not overlap with the existing nature reserves.

## 3 RESULTS

### 3.1 Model results

The AUC for the Maxent model for *F. kwangsiensis* was 0.995 (±0.004), higher than 0.5 of the random model. Precipitation in the warmest quarter (Bio18) was the most significant factor in the model, followed by precipitation in the driest quarter (BIO17), population density in 2000 (2000), annual precipitation (BIO12), temperature seasonality (BIO4), mean temperature in the wettest quarter (BIO8), the minimum temperature in the coldest month (Bio6), and the percent tree cover (PTC; Table 1). The cumulative contribution of these eight factors for *F. kwangsiensis* distribution was 98.3%.

### 3.2 Predicted current potential distribution

**FIGURE 2:**
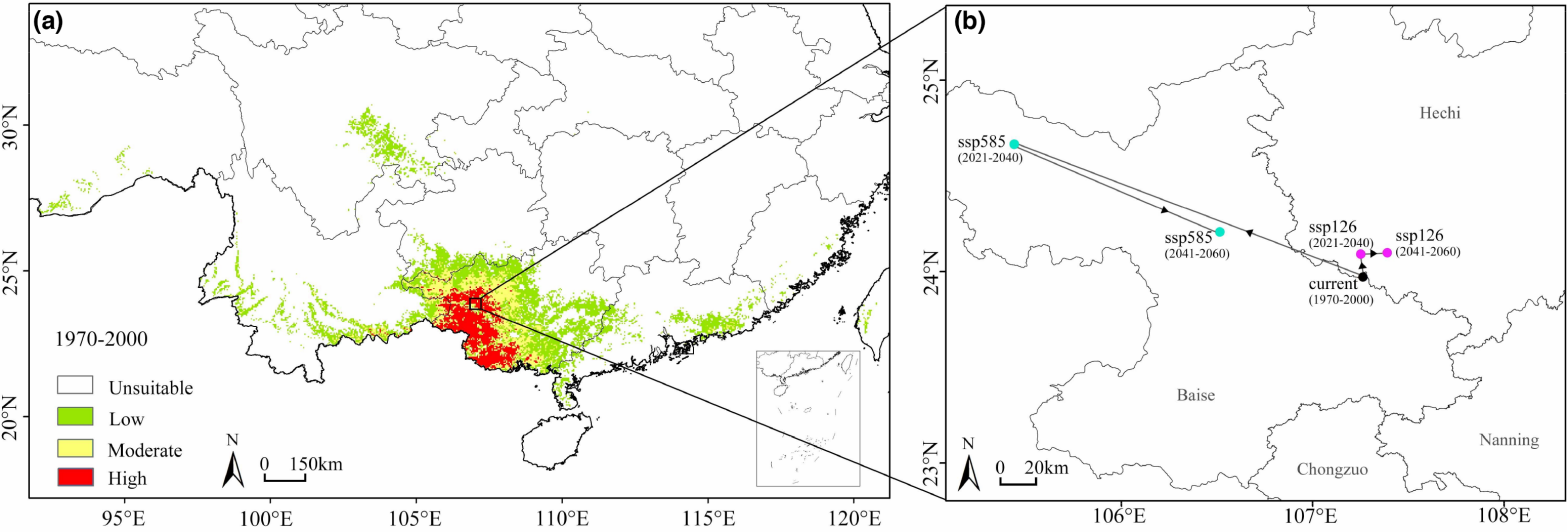
Potentially suitable habitats for *Firmiana kwangsiensis* in contemporary (a) and the distribution center migration (b) based on Maxent.

The current most suitable habitats for *F. kwangsiensis* in southwest Guangxi, where the tree species already exist were predicted. The most suitable habitat for the tree species in the southern Yunnan province, where the tree species is known to be absent, was also predicted. Notably, the current potential distribution of *F. kwangsiensis* is significantly larger than the actual occurrence of the tree, including in southern Tibet, southeast Sichuan, southeast Guangdong, southwest Guizhou, Guangxi, Fujian and Taiwan Province (Figure 2). The total current potential habitats for the tree in China were predicted to be 259,504 km^2^, with high suitable habitats accounting for 15.81% of this (Table 2).

### 3.3 Predicted future potential distribution

**FIGURE 3:**
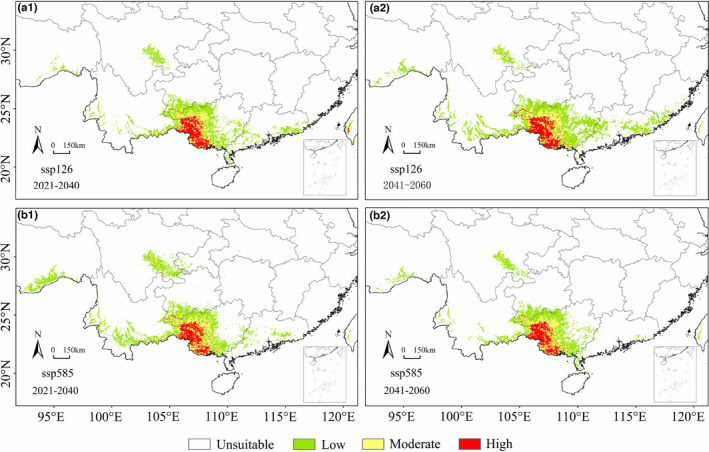
The potential distribution map of *Firmiana kwangsiensis* in 2020–2040 and 2041–2060 under the SSP126 scenario (a1, a2); the potential distribution map of *F. kwangsiensis* in 2020–2040 and 2041–2060 under the scenario of SSP585 (b1, b2).

Compared with the current potential habitat, the total potential habitat under the SSP126 scenario in 2021–2040 will reduce by 13.74%, equivalent to 223,844 km^2^. The high suitable habitat will reduce by 37,301 km^2^, 9.08% less than the current potential distribution (Table 2). The high suitability habitats are in Guangdong and Taiwan provinces, while moderate suitability will increase in Sichuan Province (Figure 3). The potential suitable distribution of *F. kwangsiensis* will expand under the SSP126 scenario in 2041–2060, whereas the moderately suitable habitat will increase in southern Fujian and central Yunnan (Figure 3). Compared with the 2021–2040 estimates, the total suitable habitat will increase by 29.46% to 289,798 km^2^, among which high suitable habitat will be 43,193 km^2^, 15.80% more than the high suitable habitat in 2021–2040 (Table 2).

Under the SSP585 scenario, the moderately suitable habitat for *F. kwangsiensis* in 2021–2040 will be in southeast Sichuan, southeast Yunnan and southern Tibet (Figure 3). The total potential habitat for the plant during this period will increase by 3.53%, whereas the high suitable habitat will decrease by 9.33% (Table 3). In the 2041–2060 period, the suitable habitat of *F. kwangsiensis* in south Tibet, southeast Sichuan, southwest Yunnan and Guangdong province will decrease (Figure 3). The total potential habitat will reduce by 22.23%, and the high suitable habitat will reduce by 4.14% (Table 2).

**TABLE 3:** The overlapped area (km^2^) between the existing protected area and the potentially suitable habitat for *Firmiana kwangsiensis*.

Protected area	Level	Area	Overlapped area
Daweishan National Nature Reserve	National	43,992.6	18.5
Malipo Laoshan Provincial Nature Reserve	Provincial	20,500.0	1.0
Nonggang National Nature Reserve	National	10,080.0	110.9
Damingshan National Nature Reserve	National	16,994.0	415.7
Shiwandashan National Nature Reserve	National	58,277.1	436.2
Xialei Provincial Nature Reserve	Provincial	27,185.0	110.8
Cenwanglaoshan Nature Reserve	National	18,994.0	40.1
Chongzuo white‐headed langur Nature Reserve	National	25,578.0	262.4
Fangcheng Golden Camellias National Nature Reserve	National	9195.1	61.1
Nazuo *Cycas* Provincial Forest Ecological Nature Reserve	Provincial	12,458.0	26.0
Huanglianshan‐Xingwang Nature Reserve	Provincial	21,035.5	50.9
Qinglongshan Nature Reserve	County	15,100.0	142.3
Bangliang National Nature Reserve	National	6530.0	62.3

The current potential distribution centroid of *F. kwangsiensis* was predicted to be in the southeast of Baise City of Guangxi (23.97°N, 107.26°E). In the SSP126 scenario, the centroid in 2021–2040 moved to the north by a short distance, and then it was offset to the east in 2041–2060. In scenario SSP585, the centroid offset greatly changed. The centroid was moved to the northwest in 2021–2040, reaching the west of Baise City. In 2041–2060, it moved to the southeast and the northwest of the contemporary centroid.

### 3.4 Assessment of conservation status

Of the 259,504 km^2^ of predicted habitat, 7016 km^2^ (2.70%) lie within the 65 existing protected areas in different categories and levels. Also, 1738 km^2^ of highly suitable habitat is located in 13 other protected areas, accounting for 4.24% of the predicted high suitable area (Table 3). The currently predicted habitat splits across the management systems, including different administrative departments (environmental protection department, agriculture department, and forestry department, among others), levels (national, provincial, and county, among others), and protection categories (forest ecology, wild plants, and wild animals, among others). The competent authorities in the highly suitable habitat are mainly forestry departments established earlier in the nature reserve areas.

## 4 DISCUSSION

### 4.1 Environmental variables affecting the distribution of *F. kwangsiensis*

The Maxent model results showed that the current high suitable habitats for *F. kwangsiensis* were mostly in southwest Guangxi. This region is characterized by high annual precipitation (1300–2000 mm) with significant seasonal variation and a mean temperature in the coldest month at >10°C. Temperature and precipitation are the most important climatic variables that limit the distribution of numerous tropical tree species (Kong et al., 2021).

It is crucial to recognize environmental factors that impact the geographical distribution of a species in terms of ecological perspective. Our results showed that BIO18 (57.5%), BIO 17 (16.8%), Population density in 2000 (6.0%), BIO 12 (5.7%), BIO4 (4.5%), BIO8 (3.1%), and BIO6 (2.4%) were the main factors affecting the distribution of *F. kwangsiensis*. The precipitation‐associated bioclimatic variables accounted for 74.30% of habitat suitability prediction of *F. kwangsiensis* and were the most robust habitat distribution predictors. Bioclimatic factors can influence the growth of plants and the survival of seedlings (Poorter & Nagel, 2000). *F. kwangsiensis* has only been observed in the subtropical monsoon climate regions with porous brown limestone soils with frequent soil water scarcity. Soil water stress can disrupt plant metabolism and change the morphology of a plant, thus limiting the growth of a plant or even causing death (Zhang et al., 2018). Therefore, *F. kwangsiensis* requires prolonged high precipitation in the growing season.

The physiological toleration hypothesis suggests that the tolerance to a specific range of temperature has often been used to explains the latitudinal distribution of a species (Stocker et al., 2013). Also, temperature‐associated bioclimatic variables are key to predicting *F. kwangsiensis* habitat suitability (Table 1)*. F. kwangsiensis* grows well in warm and humid areas where the temperature seasonality is ca. 5°C, and the mean temperature in the coldest month is >8°C. This temperature in accordance with the identified climatic preference for *F. kwangsiensis* (Luo et al., 2011). Temperature change impacts the physiological metabolism activities such as respiration, photosynthesis and water absorption, of *F. kwangsiensis*, influencing the distribution of this tree species. The high temperature in summer may affect the seed germination and seedling growth of *F. kwangsiensis* (Baskin & Baskin, 2014). The field investigation showed that *F. kwangsiensis* does not grow in cold regions with a mean temperature of <0°C. Therefore, we concluded that *F. kwangsiensis* grows best in moisture and high‐temperature climate regions, attributed to its tropical adaptation.

SDM generally represents the fundamental niches of species but not the actual niches (Falk & Mellert, 2011). Numerous factors, such as interspecific competition and plant–animal interactions that influence the dimension of a niche, are not considered when predicting the potential geographical distributions of many species (Deb et al., 2017a). In a study of the metabolic rate of 14 passerine species in the northern boundary of the United States and Canada, Root (1988) found the average temperature in January in the northern boundary of the region where the Eastern Phoebe (*Sayornis phoebe*) grows is −4°C. Further study indicated that the winter distribution of this avian species is directly linked to food availability. According to Huang et al. (2018), sunbirds were the major pollinators of *F. kwangsiensis*. Considering the northern boundary of sunbirds' distribution does not go beyond 34°N (MacKinnon, 2021), our prediction of the potential distribution of *F. kwangsiensis* is reliable. However, certain parameters such as species' dispersal abilities, interspecific competition, and geographical obstruction that influence the distribution of *F. kwangsiensis* were not considered for lack of available data. As a result, future research should explore the contribution of these parameters (Elith et al., 2006).

### 4.2 Predicted potential distribution of *F. kwangsiensis*

Increasing evidence shows that the global average temperature is increasing, partially in response to the increased emission of greenhouse gases (Muñoz et al., 2013). The distribution of many species has been influenced by global climate change. However, the response differs among species. For example, Zhang et al. (2021) found the distribution of *Xanthium italicum* is expected to shrink in the future, attributed to climate change. Separate research revealed that climate change positively impacts the distribution of four of the six species (Della Rocca & Milanesi, 2020). Maxent model revealed that highly suitable habitats of *F. kwangsiensis* are expected to reduce in 2021–2040. However, under SSP126, the *F. kwangsiensis* is predicted to increase in 2041–2060, whereas high suitable habitats of *F. kwangsiensis* are expected to decrease over time (Table 2). Climate change may cause local extinction of *F. kwangsiensis* and related species in some areas. However, *F. kwangsiensis* may adapt and grow in new habitats (Deb et al., 2017a). Changes in precipitation rate, duration, and temperature may cause phenological shifts in *F. kwangsiensis*, directly affecting the distribution of floral and faunal that interact with *F. kwangsiensis* at some point of their lifecycles. In addition, climate change will negatively affect the distribution of numerous insects, birds, and mammals that indirectly depend on this tree species (Deb et al., 2017b).

### 4.3 Conservation of *F. kwangsiensis*

**FIGURE 4:**
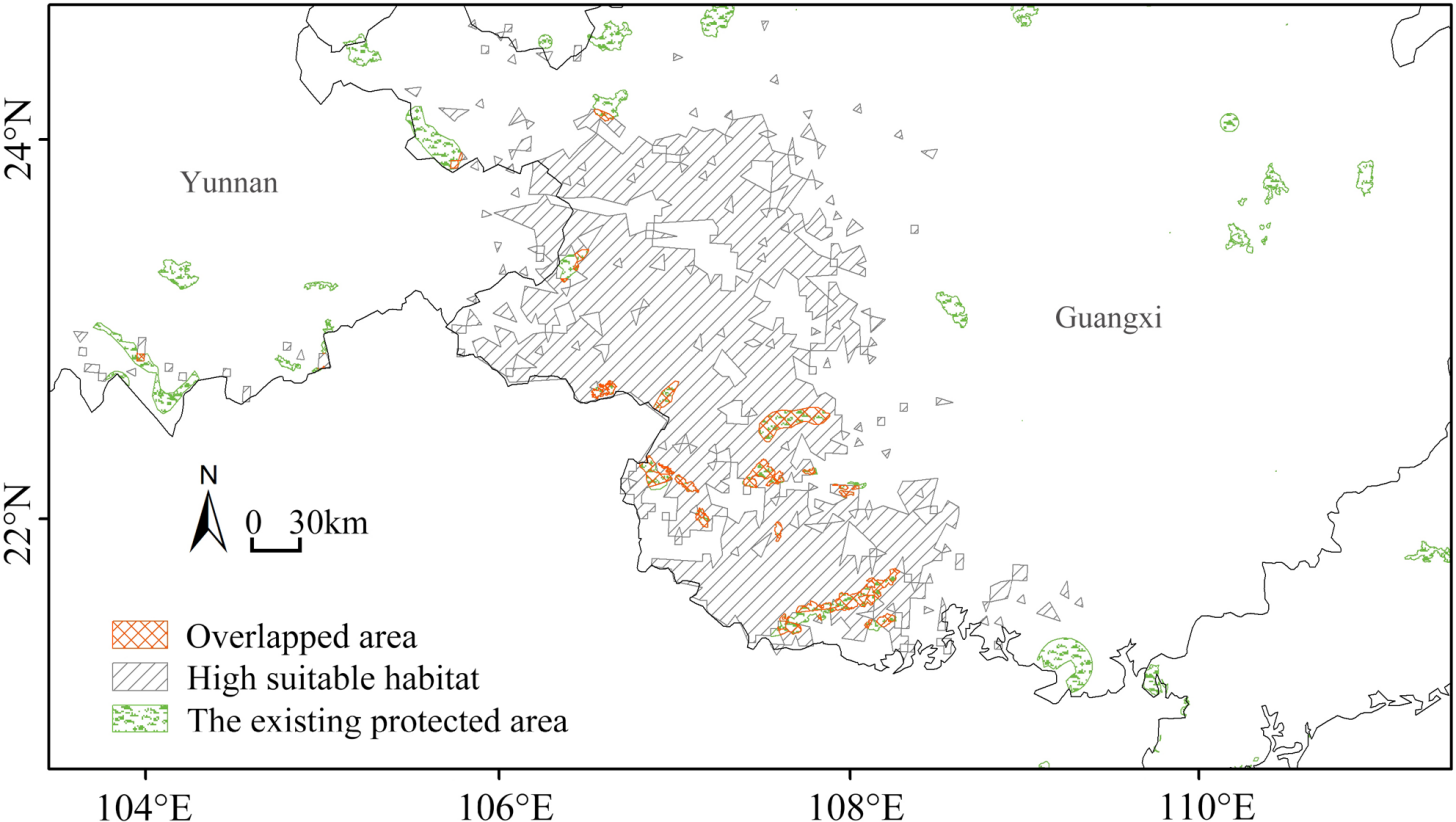
The overlapped area between high suitable habitat and the existing protected area for *Firmiana kwangsiensis*.

The existing *F. kwangsiensis* cover is only 2.70% (7016 km^2^) of potential habitats and 4.24% (1738 km^2^) of high potential habitats (Figure 4). By contrast, *F. kwangsiensis* covers 7.17% of the potential habitat in Guangxi. Therefore, the *F. kwangsiensis*'s habitat in Guangxi was far less than the average area for the wild plants and animals in the area. While protected areas have proved effective in protecting endangered species from ongoing threats from human activities, many species are likely to shift their distributions outside the protected areas under climate change scenarios (Kong et al., 2021; Thapa et al., 2018). At the same time, the anthropogenic climate change can reduce the ability of plants to shift their ranges by changing the spatial distribution of faunal that interact with the plants (McConkey et al., 2012). The habitat fragmentation and other land‐use changes will likely amplify existing constraints on plant range shifts by hampering pollination and seed dispersal (Tucker et al., 2018). This underscores the need to not only establish more natural small protected areas to maximize protection of this endangered species but also restore biotic connectivity through the recovery of animals to increase the resilience of vegetation communities under climate change (Fricke et al., 2022). This means that most of *F. kwangsiensis* habitats are currently non‐protected, indicating the growing effect of anthropogenic activities and climate change on the distribution of this species. Accordingly, we suggest that *F. kwangsiensis* conservation should not only rely on existing nature reserves but also include small protected‐area and wildlife corridors.

In this study, we explored the consequences of climate change on the geographical distribution of F. kwangsiensis, an endangered species easily recognized in the field, to provide a reference for establishing protected areas in its potential habitat. However, data on a single species is insufficient to delimit the boundaries of protected areas. Accordingly, more similar studies, especially the potential distribution of pollinators and seed dispersers, are needed further to inform the most suitable protected areas for *F. kwangsiensis*. At the same time, limited resources and human resources are the major constraints of species conservation. Therefore, it is necessary to identify priority protected areas for endangered species, and carry out small‐scale conservation planning for the inadequate protected areas.

The findings of this study can inform the formulation of the conservation guidelines for *F. kwangsiensis*. In general, there is a need to establish *F. kwangsiensis* protected areas and expand the existing reserves in the high‐risk regions in the contest climatic change. Our analysis further identified suitable habitats for ex situ conservation of *F. kwangsiensis*. Furthermore, areas without a change in *F. kwangsiensis* distribution in the face of climate change are possible climate change refugia regions.

## AUTHOR CONTRIBUTIONS

**Xiao‐Xuan Gao:** Conceptualization (equal); data curation (equal); formal analysis (equal); investigation (equal); methodology (equal); writing – original draft (equal). **Jing Liu:** Conceptualization (equal); data curation (equal); formal analysis (equal); investigation (equal); methodology (equal). **Zhi‐Huan Huang:** Conceptualization (equal); data curation (equal); formal analysis (equal); funding acquisition (lead); investigation (equal); methodology (equal); writing – original draft (equal).

## CONFLICT OF INTEREST

The authors declare no competing interests.

## Data Availability

The data that support the findings of this study are available on request from the corresponding author. The data are not publicly available due to privacy or ethical restrictions.
